# Targeting Cancer Metabolism to Resensitize Chemotherapy: Potential Development of Cancer Chemosensitizers from Traditional Chinese Medicines

**DOI:** 10.3390/cancers12020404

**Published:** 2020-02-10

**Authors:** Wei Guo, Hor-Yue Tan, Feiyu Chen, Ning Wang, Yibin Feng

**Affiliations:** School of Chinese Medicine, Li Ka Shing Faculty of Medicine, The University of Hong Kong, Hong Kong SAR 00000, China

**Keywords:** cancer metabolism, metabolic reprogramming, chemoresistance, traditional chinese medicines, chemosensitizer

## Abstract

Cancer is a common and complex disease with high incidence and mortality rates, which causes a severe public health problem worldwide. As one of the standard therapeutic approaches for cancer therapy, the prognosis and outcome of chemotherapy are still far from satisfactory due to the severe side effects and increasingly acquired resistance. The development of novel and effective treatment strategies to overcome chemoresistance is urgent for cancer therapy. Metabolic reprogramming is one of the hallmarks of cancer. Cancer cells could rewire metabolic pathways to facilitate tumorigenesis, tumor progression, and metastasis, as well as chemoresistance. The metabolic reprogramming may serve as a promising therapeutic strategy and rekindle the research enthusiasm for overcoming chemoresistance. This review focuses on emerging mechanisms underlying rewired metabolic pathways for cancer chemoresistance in terms of glucose and energy, lipid, amino acid, and nucleotide metabolisms, as well as other related metabolisms. In particular, we highlight the potential of traditional Chinese medicine as a chemosensitizer for cancer chemotherapy from the metabolic perspective. The perspectives of metabolic targeting to chemoresistance are also discussed. In conclusion, the elucidation of the underlying metabolic reprogramming mechanisms by which cancer cells develop chemoresistance and traditional Chinese medicines resensitize chemotherapy would provide us a new insight into developing promising therapeutics and scientific evidence for clinical use of traditional Chinese medicine as a chemosensitizer for cancer therapy.

## 1. Introduction

Cancer is a common and complex disease with various genetic and environmental risks. The mortality rate of cancer is similar to its incidence rate, and both rates are markedly increasing year by year, causing severe public health problems worldwide. There were approximately 18.1 million new cases of cancers and 9.6 million cancer-related deaths all over the world in 2018 [1]. The number of patients diagnosed of cancer reached almost 14.5 million in 2014 and is expected to rise to nearly 19 million by 2024 [2]. More than 60% of the new cases of cancer worldwide appear in Asia, Africa, and Central and South America.

Meanwhile, nearly 70% of cancer-related deaths worldwide also appear in these areas. For both males and females, lung cancer is the most common cancer with the highest incidence and mortality rates, closely followed by colorectal cancer for both rates. As for males, lung cancer with the most common cancer incidence is the leading cause of cancer-related death, followed by prostate and colorectal cancer (for incidence) and liver and stomach cancer (for mortality). As for females, breast cancer with the most common cancer incidence is the leading cause of cancer-related death, followed by colorectal and lung cancer for both rates. The current therapeutics for cancer mainly consist of three conventional therapies: surgery, chemotherapy, and radiotherapy. In addition to the conventional therapies, there are also several modern therapies, such as biologically targeted therapy, immunotherapy, and hormone therapy [3]. Among these therapies for cancer, surgery is the only potentially curative option. However, surgical resection is not suitable for most cancer patients with an advanced state due to the late diagnosis [4]. To some degree, the modern therapies only apply to early and certain kinds of cancer, and are not desirable for the majority of cancer in middle-late stages [5]. Non-surgical approaches such as chemotherapy and radiotherapy remain the standard therapeutic approaches for cancer patients [6].

There are more than 200 anticancer drugs and they could be allocated into eight classes according to their action of mechanisms: alkylating drugs, mitotic inhibitors, topoisomerase inhibitors, antitumor antibiotics, antimetabolites, hormones and antagonists, corticosteroids, and biologically targeted drugs such as tyrosine kinase inhibitors (TKIs), phosphoinositide 3-kinase (PI3K)/protein kinase B (AKT)/mammalian target of rapamycin (mTOR) inhibitors, and other pathway inhibitors [2]. As the most frequently used anticancer drugs, chemotherapeutic drugs including alkylating drugs, mitotic inhibitors, topoisomerase inhibitors, antitumor antibiotics, and antimetabolites predominantly work on the induction of cell death, such as premature senescence, mitotic catastrophe, and apoptosis. Although the abundant types of chemotherapeutic drugs are used for cancer therapy, the prognosis and outcome of chemotherapy are still far from satisfactory. Besides severe side effects, the treatment may develop resistance and chemoresistance was considered as the primary clinical obstacle to the successful cure for cancer [7]. Cancer cells could gradually develop either intrinsic or acquired resistance to almost all chemotherapeutic drugs. What is more, cancer cells could not only show chemoresistance to a single drug to which they are exposed, but also could generate cross-resistance to other multiple agents [8]. Therefore, it is urgently needed to investigate the underlying molecular mechanisms of chemoresistance and further develop novel and effective treatment strategies to overcome chemotherapy resistance for cancer therapy.

Metabolic reprogramming is one of the hallmarks of cancer [9]. Cancer cells could rewire metabolic pathways to support their rapid proliferation by increased adenosine triphosphate (ATP) generation, macromolecule synthesis, and antioxidant regeneration [10]. Metabolic reprogramming redefines the flow and flux of metabolites in cancer cells in terms of the metabolic network, which is mediated by various tumor suppressor genes and oncogenes. Cancer metabolism provides valuable information for tumorigenesis, tumor progression, and metastasis, which could contribute to the identification of new and useful biomarkers for cancer diagnosis and novel targets for cancer therapy [11]. Recently, cancer metabolism has become a hot spot for cancer research.

With the increasing understanding of the mechanisms by which cancer cells develop chemoresistance, it has been suggested that various mechanisms are involved in this process, such as variations in drug metabolism, mutations in drug targets, changes in DNA repair, formation of cancer stem cells (CSCs), immunosuppression, inactivation of apoptotic genes, and activation of anti-apoptotic genes [12]. Recently, the relationship between cancer metabolic reprogramming and chemoresistance has been suggested [13,14]. Of note, the chemo-resistant cancer cells could rewire their metabolic pathways, which contributes to drug-induced adaptive resistance. The metabolic reprogramming may serve as a promising therapeutic strategy and rekindle the research enthusiasm for overcoming chemotherapy resistance [15].

Traditional Chinese medicine has been used in the therapy for various diseases, including cancer, over thousands of years in Asian countries, especially in China and Japan. Traditional Chinese medicine has also been gradually accepted as a complementary and alternative approach in western countries [16]. Increasing studies have suggested that a combination of traditional Chinese medicine with conventional chemotherapies has the great potential to improve the chemotherapy response via multiple molecular mechanisms [17,18]. Based on the close relationship between cancer metabolic reprogramming and chemoresistance mentioned above, cancer metabolism would be considered as a promising therapeutic strategy for overcoming chemotherapy resistance by targeting multiple cancer metabolisms. The “multi-components and multi-targets” manifestations of traditional Chinese medicines make them an attractive direction for developing new adjuvant therapeutics to reverse chemoresistance. Many compounds derived from traditional Chinese medicine could reverse chemotherapeutic drug resistance by metabolic regulation. Traditional Chinese medicine is considered as a neoadjuvant to resensitize chemotherapy from the metabolic perspective, which would open up new avenues for overcoming chemotherapy resistance.

**Figure 1:**
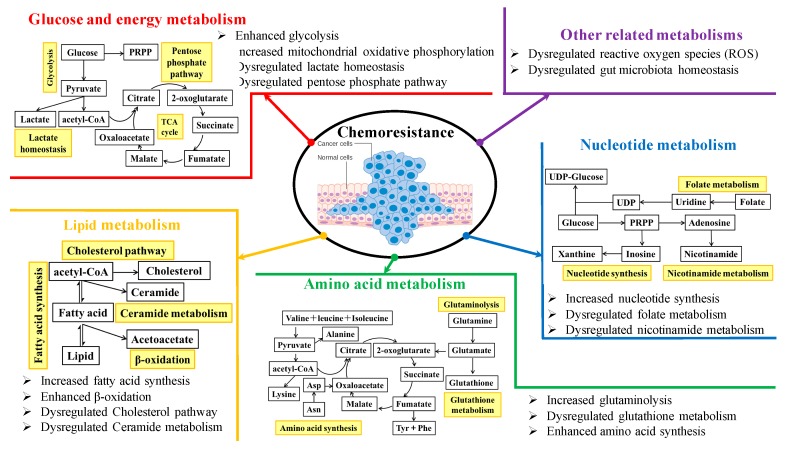
An overview of chemoresistance modulated by metabolic reprogramming in cancer cells.

We systematically searched among the database PubMed using the five keywords “cancer metabolism”, “metabolic reprogramming”, “chemoresistance”, “Chinese medicine”, and “chemosensitizer”. The emerging molecular mechanisms underlying rewired metabolic pathways for cancer chemoresistance are discussed in terms of glucose and energy, lipid, amino acid, and nucleotide metabolisms, as well as other related metabolisms. Figure 1 shows an overview of chemoresistance modulated by metabolic reprogramming in cancer cells. The perspectives of metabolic targeting to chemoresistance are also discussed. In particular, as an alternative therapeutic option, the present use of traditional Chinese medicine in combination with chemotherapeutics for overcoming resistance from the metabolic perspective is highlighted. Overall, the elucidation of the underlying metabolic reprogramming mechanisms by which cancer cells develop chemoresistance and traditional Chinese medicine resensitizes chemotherapies would provide us a new insight into the strategies of discovery and development of promising cancer chemosensitizers from clinically used traditional Chinese medicine.

## 2. Metabolic Targeting to Sensitize Cancer Chemotherapy

Cancer metabolic reprogramming is a common phenomenon associated with tumorigenesis, tumor progression, and metastasis, as well as chemoresistance. Generally speaking, the metabolic modifiers could aim to regulate the enzymes and other related factors such as transporters involved in cancer metabolism to suppress the ATP production from cancer glucose consumption and attenuate the synthesis of fatty acid, amino acids, and nucleotides, all of which could be considered as the promising therapeutic targets for overcoming chemotherapy resistance via metabolic intervention.

### 2.1. Glucose and Energy Metabolism

Increasing evidence has revealed that cancer cells rewire metabolic pathways, primarily glucose and energy metabolism, to support their rapid proliferation. It has been shown that normal cells generate energy mainly via tricarboxylic acid (TCA) cycle-sustained mitochondrial oxidative phosphorylation (OXPHOS), while cancer cells produce energy mostly via aerobic glycolysis, which was defined as a well-known phenomenon called “Warburg effect” [19]. In the hypoxia and hypo-nutrient microenvironment, cancer cells enhance glycolytic pathway to generate ATP as the main source for energy production, which is controlled by the interaction between the oxygen-sensing transcription factor and the nutrient-sensing signal pathway [20,21]. Adenosine monophosphate-activated protein kinase (AMPK), a sensor of energy status, is intimately involved in Warburg effect [22]. In detail, the inactivation of AMPK by genetic ablation induces the normoxic stabilization of hypoxia-inducible factor-1alpha (HIF-1α) and confers the proliferative advantages of cancer cells accompanied by the metabolic shift to aerobic glycolysis [23]. First, as the main process for cancer cells to produce energy, the relationship between glycolysis and the occurrence of chemoresistance has been systematically demonstrated. For example, compared with normal B cells, chronic lymphocytic leukemia (CLL) cells were reported to have modified energy metabolism. To investigate the relationship between the molecular and metabolic heterogeneity and characterize the role of energy metabolism in response to drug treatments, Lu et al. analyzed metabolic flux and estimated the bioenergetic features of samples from 140 primary CLL patients [24]. They found that increased glycolytic activity resulted in heightened resistance of mitochondria targeting drugs, including orlistat, navitoclax, rotenone, and venetoclax, which was controlled by the genomic markers the immunoglobulin (Ig) heavy chain (IGHV) genes and TP53. Gemcitabine is the principal chemotherapy agent for the treatment of pancreatic cancer. L-type amino acid transporter 2, a neutral amino acid transporter, was found to decrease the chemosensitivity of gemcitabine to pancreatic cancer, which was mediated by increased glycolysis via glutamine-dependent mTOR activation [25]. The high expression of hexokinase 2 (HK2) was also found to be associated with gemcitabine resistance to pancreatic cancer, which is the core enzyme to regulate glycolysis by the first-step reaction [26]. Besides pancreatic cancer, a high level of HK2 was also related to the chemoresistance in epithelial ovarian cancer [27]. Glucose transporter type 1 (GLUT1) is the essential transporter to shift glucose from the extracellular environment to the cells. It has been suggested that inhibition of GLUT1 by either RNA interference or N4-[1-(4-cyanobenzyl)-5-methyl-3-(trifluoromethyl)-1H-pyrazol-4-yl]-7-fluoroquinoline-2,4-dicarboxamide (BAY-876, a specific GLUT1 inhibitor) could resensitize cisplatin to esophageal squamous cell carcinoma (ESCC) [28]. High level of GLUT1 also decreased the responsiveness of acute myeloid leukemia (AML) to adriamycin [29], which could be a potential target to overcome chemoresistance. Pyruvate dehydrogenase kinase 1 (PDK1) is an essential enzyme for glucose metabolism. A recent study has reported that the high expression level of PDK1 played an obligate role in the resistance of cisplatin chemotherapy for ovarian cancer [30]. Maurer et al. reported that the TP53-induced glycolysis and apoptosis regulator (TIGAR) regulated glycolysis and induced chemoresistance of glioblastoma to temozolomide, which was mediated by HIF-1α [31]. In tumor, cancer cells coexist with multiple non-neoplastic stromal cells, together creating the tumor microenvironment. Recently, the cross-interaction between cancer cells and carcinoma-associated fibroblasts (CAFs) has been suggested [32]. In detail, CAFs produced energy metabolites to create the necessary energy-rich microenvironment for facilitating tumor growth through higher aerobic glycolysis (reverse Warburg effect) and autophagy, which was associated with the loss of stromal caveolin-1 (Cav-1) expression [33,34]. Remarkably, Sotgia et al. reported that the activation of aerobic glycolysis and autophagy of breast CAFs via the down-regulation of Cav-1 also accounted for the poor clinical outcome in breast cancer patients treated with tamoxifen [35].

Lactate production, the essential pathway related to glycolysis, was also reported to be closely associated with the acquired chemoresistance. For example, the monocarboxylate transporter (MCT) 1-mediated lactate homeostasis by lactate transportation played an essential role in the resistance of breast cancer to paclitaxel, which was regulated by microRNA-124 [36]. Lactate dehydrogenase (LDH), as the essential enzyme for shifting pyruvate to lactate to produce ATP, is of primary importance in glucose metabolism. Zhang et al. reported that the high aerobic glycolysis flux decreased the chemosensitivity of leukemia to adriamycin, which was regulated by the AKT-mTOR pathway and could be reversed by the LDH inhibitor oxamate [37]. As one of the frequently used chemotherapeutic drugs for prostate cancer, the resistance of docetaxel was revealed to be regulated by L-lactate dehydrogenase A (LDHA)-mediated lactate homeostasis [38]. Interestingly, the synergistic antineoplastic property was found by the combination therapy of docetaxel and sodium oxamate, a specific LDHA inhibitor. Zhou et al. also found that the high level of LDHA was associated with taxol resistance to breast cancer, which could be reversed by the specific LDH inhibitor oxamate [39]. As another isoform of LDH, L-lactate dehydrogenase B (LDHB), was revealed to be the sensitive biomarker for the acquired chemoresistance of cetuximab to colorectal cancer [40]. Mesenchymal-epithelial transition tyrosine kinase receptor (MET)/epidermal growth factor receptor (EGFR) TKIs were commonly used for cancer chemotherapy. However, the increased resistance to TKIs decreased their effects. A recent study revealed that the resistance of TKIs in multiple cancers was relevant to increased lactate production by activated LDH, MCT4, and MCT1, the inhibition of which could abrogate the resistant properties of MET/EGFR TKIs [41].

As a branch of the glycolysis pathway, the pentose phosphate pathway (PPP) plays an important role in cancer cell proliferation by linking glucose metabolism and nucleotide synthesis. Recently, the relationship between PPP and chemoresistance has been characterized. Taxanes induced adaptive tolerance to breast cancer cells, which was associated with metabolic transition characterized by elevated glucose flux of both glycolytic and oxidative respiration via the PPP [42]. Glucose-6-phosphate dehydrogenase (G6PD) played an important role during this metabolic transition. You et al. conducted a combined stable isotope labeling mass spectrometry and systematic untargeted metabolomics analysis to characterize the metabolic reprogramming associated with the resistance of sorafenib to AML [43]. They found that sorafenib resistant cancer cells inhibited a reduced glucose flux into the PPP, which was mediated by the enzymes G6PD and transketolase. Pyruvate kinase M2 (PKM2), an essential enzyme involved in glucose metabolism, was revealed to decrease the sensitivity of cisplatin to ESCC by activating PPP [44].

Besides aerobic glycolysis, the mitochondrial OXPHOS also played an essential role in the chemotherapy resistance. For example, the essential mitochondrial dysfunction in energy production was suggested to account for the resistance of several chemotherapeutic drugs in breast cancer, such as cisplatin, doxorubicin, and cyclophosphamide. Of note, this mitochondrial dysfunction could be reversed by bactericidal antibiotics linezolid, suggesting that specific antibiotics may be regarded as a new therapy for cancer [45]. Zhuang et al. found that the diminished activity of pyruvate dehydrogenase could decrease mitochondrial oxygen consumption and thus resulted in the resistance of metformin chemotherapy to breast cancer, which was mediated by the tumor protein D54 [46]. High level of intracellular ATP induced by aerobic glycolysis and defective mitochondrial ATP production could contribute to the chemoresistance of multiple chemotherapeutic agents in colon cancer, which was mediated by HIF-1α signaling [47]. The v-raf murine sarcoma viral oncogenes homolog B1 mutation (BRAF^V600E^) inhibitors used for the treatment of melanoma lose their efficacy due to the acquisition of drug resistance. In detail, the inhibition of BRAF induced an adaptive metabolic shift to mitochondrial metabolism by increasing the mitochondrial master regulator-peroxisome proliferator-activated receptor gamma coactivator-1 alpha (PGC-1a) and the melanocyte lineage factor-microphthalmia-associated transcription factor (MITF) [48], which could be developed as novel therapeutic strategy [20]. Notably, suppression of mitochondrial respiration resensitized the therapy of melanoma cells via hindering the emergence of the highly expressed histone 3 K4 demethylase (JARID1B^high^) subpopulation [49].

Various compounds derived from traditional Chinese medicine targeting the glucose and energy metabolism could reverse chemotherapeutic agent-induced resistance. For example, baicalein is a flavonoid isolated from the plant *Scutellaria baicalensis* with strong antineoplastic properties. A recent study revealed that baicalein could reverse 5-fluorouracil induced resistance in gastric cancer via inhibiting the phosphatase and tensin homolog deleted on chromosome 10 (PTEN)/AKT/HIF-1α mediated glycolysis [50]. Deguelin, derived from several medicinal plants, is a mitochondrial complex I inhibitor. It was shown that deguelin could inhibit oxygen consumption via mitochondrial OXPHOS and thus resensitize the resistance of vemurafenib to melanoma, which was mediated by activated AMPK signaling [51]. Trichostatin A, as an active compound isolated from *Streptomyces hygroscopicus*, is a potent histone-deacetylase inhibitor. Based on the transcriptomics and metabolomics analyses, a recent study revealed that the combination therapy of trichostatin A could enhance sunitinib sensitivity against renal cell carcinoma by triggering intracellular metabolome shifts in terms of energy metabolism [52]. Sodium selenite is a kind of mineral. It has been suggested that sodium selenite could significantly resensitize the chemoresistant human bladder cancer cell line RT-112/D21 by altering mitochondrial functions [53]. Furanodiene is an active sesquiterpene derived from the plant *Curcuma wenyujin* with potential anti-tumor effects. A recent study showed that furanodiene also altered mitochondrial functions to resensitize the effects of doxorubicin on breast cancer [54]. Rhein is a monomeric anthraquinone derived from two plant herb species *P. cuspidatum* and *Polygonum multiflorum* with strong anti-cancer effects. A recent study has found that rhein could reverse doxorubicin-induced resistance in liver cancer primarily by inhibiting mitochondrial energy metabolism [55]. Apart from the pure compounds from traditional Chinese medicine, the Chinese medicinal prescriptions are also indicated to improve the poor responsiveness of chemotherapy. For example, the Dahuang Zhechong Pill (DHZCP), as a classical, traditional Chinese medicinal prescription, has a long history of clinical use for liver cancer therapy. Recently, both in vitro and in vivo effects of DHZCP in potentiating doxorubicin to liver cancer were revealed. In detail, DHZCP markedly decreased the ATP level by suppressing the critical enzymes involved in TCA cycle and OXPHOS to reverse the doxorubicin-induced resistance in liver cancer [56,57]. Table 1 summarizes the recent studies on combination therapy of chemotherapeutic agents and traditional Chinese medicines to overcome chemoresistance targeting glucose and energy metabolism.

### 2.2. Lipid Metabolism

Besides glucose and energy metabolism, cancer cells could also rewire their lipid metabolic reprogramming for their rapid proliferation by both de novo lipogenic synthesis and lipid catabolic metabolisms. Notably, in some nonglycolytic cancers, such as lymphoma and prostate cancer, lipid-dependent metabolism could become the predominant pathway to produce energy, which facilitates the rapid growth and metastasis of cancer cells [58,59]. Cancer cells even store excessive energy in the form of lipid droplets, which could be further broken down into free fatty acids to supply energy, similar to adipocytes [60]. Recently, the close relationship between lipid metabolic reprogramming and cancer drug resistance has also been suggested [61]. The mechanisms involved in the chemoresistance through lipid-dependent metabolic reprogramming were systematically reviewed.

OMP52M51, as a notch1-specific neutralizing antibody, was reported to show anti-tumor effects on T-cell acute lymphoblastic leukemia. However, the acquired resistance of OMP52M51 eventually developed and disappointed its efficacy, which was accompanied by the regulation of lipid metabolism [62]. G protein-coupled receptor 120 (GPR120) is a receptor for long-chain fatty acids. It has been reported that the resistance of breast cancer cells to epirubicin was induced by the increasing de novo synthesis of fatty acids via GPR120 [63]. The resistance of the antiangiogenic drugs (AAD) is commonly developed in cancer patients and disappoints the treatments. Iwamoto et al. found that AAD resistance could be evoked by lipid-dependent metabolic reprogramming. In detail, the tumor hypoxia caused by AAD triggered the metabolic reprogramming of fatty acid oxidation to support the rapid proliferation of cancer cells. In particular, suppression of carnitine palmitoyltransferase 1A via both genetic and pharmacological inhibition could increase the anti-tumor effects of AAD and resensitize their therapeutic efficacy primarily through compromising the free fatty acid-induced cell proliferation [64]. Lipid metabolism has an essential role in epithelial–mesenchymal transition, which is closely related to the development of drug resistance. Jin et al. reported that simvastatin, a cholesterol-lowering drug, could resensitize lung cancer cells to paclitaxel through lipid metabolism via disrupting the lipid rafts and suppressing the focal adhesion kinase (FAK) signaling pathway [65]. 3-hydroxy-3-methyl-glutaryl-CoA reductase (HMGCR) is a key enzyme involved in the mevalonate pathway for cholesterol biosynthesis. Kong et al. reported that HMGCR was increased in the enzalutamide-resistant prostate cancer cells. Specifically, the resistance of prostate cancer cells to enzalutamide could be reversed by the combined treatment of simvastatin, which is an inhibitor of HMGCR [66]. It was also reported that the elevated level of cholesteryl ester via acyl-CoA cholesterol acyltransferase-1 (ACAT-1) contributed to the resistance of pancreatic ductal adenocarcinoma (PDAC) cells to gemcitabine. Of note, avasimibe, an essential inhibitor of ACAT-1, could effectively resensitize PDAC cells to gemcitabine [67]. The formation and secretion of sphingosine-1-phosphate via sphingosine kinase 1 could contribute to the resistance of breast cancer cells to tamoxifen [68]. Ceramide, a kind of sphingolipid, was reported to have an important role in response to chemotherapy via cancer cell apoptosis. The lipidomic analysis conducted by Kao et al. revealed that the selection pressure of daunorubicin and cytarabine, specific drugs for treating of AML, could induce a pronounced deficiency in ceramide level and high abundance in levels of ceramide 1-phosphate and sphingosine 1-phosphate, which further evoked mitochondrial remodeling and contributed to chemoresistance [69]. It was also reported that the chemosensitivity of colon cancer cells to 5-fluorouracil or oxaliplatin could be increased by reduced autophagy and mitochondrial respiration via the downregulation of ceramide synthase 5 expression [70].

The possible approaches for overcoming chemoresistance by a combination of traditional Chinese medicines have been suggested through the regulation of lipid metabolism. For example, as an aglycone of ginsenosides separated from *Panax ginseng*, 20 (S)-protopanaxatriol (g-PPT) was reported to show multiple bioactivities, such as anti-metabolic and anti-cancer properties. Notably, it is an effective lipid metabolism inhibitor. A recent study revealed that g-PPT could markedly suppress the expression of lipid metabolism key enzyme stearoyl-CoA desaturase 1 (SCD1) and then lower the intracellular lipid droplet content in non-small cell lung cancer (NSCLC). The co-administration of g-PPT and gefitinib could reverse the EGFR TKIs resistance by inhibiting the SCD1 triggered lipid accumulation in NSCLC [71]. Melittin, a water-soluble toxic peptide isolated from bee venom, is a famous traditional Chinese medicine commonly used for the treatment of immunological diseases, chronic inflammation, and cancers. Wang et al. reported that melittin could suppress the tumor growth of PDAC by markedly mediating the cholesterol biosynthesis pathway. Of note, melittin could also reverse the resistance of PDAC cells to gemcitabine by suppressing the expression of cholesterol pathway gene clusterin [72].

### 2.3. Amino Acid Metabolism

Instead of the consumption of glucose, cancer cells have also been shown to favor glutamine as a preferential fuel. Glutamine is metabolized by a process chartered as glutaminolysis. In this process, glutamine is converted to glutamate and subsequently to α-ketoglutarate for energy production [73]. Glutaminolysis metabolism plays a vital role in the tumorigenesis and the occurrence of chemoresistance. For example, Obrist et al. conducted mass spectrometric metabolomics and specific interventions on glutamine metabolism and found that glutamine was majorly utilized for nucleotide biosynthesis rather than for bioenergetic, anaplerotic or redox reactions in cisplatin-resistant NSCLC and ovarian cancer cells [74]. The reprogramming of glutaminolysis via the Fbxo4-cyclin D1 axis was reported to contribute to CDK4/6 inhibitor resistance in human ESCC [75]. Disruption of glutamine metabolic pathways could enhance the drug responsiveness of pancreatic cancer to gemcitabine treatment [76]. Platinum-resistant ovarian cancer cells exhibited a pronounced dependency on glutamine, which was characterized by the enriched expression of the glutaminase and glutamine transporter [77]. C-Myc oncogenic transcription factor (c-Myc) was indicated to increase the glutaminase mediated glutamine catabolism and contribute to tumorigenesis through a widespread repression of miRNA expression, especially miR-23a and miR-23b [78,79]. Of note, Yoshida et al. reported that c-Myc-induced epigenetic reprogramming of CSCs was responsible for the poor responsiveness to chemotherapy [80]. Besides the critical metabolite glutamine, it has been indicated that many other amino acid metabolisms also have an essential role in the occurrence of chemoresistance. For instance, Shi et al. reported that the four enzymes associated with glutathione metabolism, namely glutathione peroxidase 4 (GPX4), aminopeptidase N (CD13), 5-oxoprolinase (OPLAH), and ribonucleotide reductase regulatory TP53 inducible subunit M2B (RRM2B), played an essential role in the chemoresistance of NSCLC to cisplatin [81]. Glutathione S-transferase alpha 1, the major glutathione S-transferase (GST) isozyme, was found to be closely related to cisplatin resistance in common types of solid cancers [82]. Yeon et al. revealed that the cisplatin resistance of bladder cancer was closely associated with argininosuccinate synthase 1 and spermidine/spermine N1-acetyltransferase-mediated amino acid metabolism [83]. Continuous sublethal EGFR-TKI treatment eventually results in TKI resistance in lung cancer. It was reported that this adaptive process was markedly related to H3K9 demethylation-mediated upregulation of branched-chain amino acid aminotransferase 1 and subsequent metabolic reprogramming [84].

An increasing amount of evidence indicates that traditional Chinese medicine could enhance the therapeutic effects of chemotherapy by targeting amino acid metabolism. For example, ursolic acid is a natural compound derived from various medicinal herbs with potential biological effects against cancer. By using ultrahigh performance liquid chromatography-tandem mass spectrometry (UPLC-MS/MS) based metabolomics, Zong et al. found that ursolic acid could reverse the resistance of doxorubicin to breast cancer. Besides the regulation of energy metabolism, including glycolysis and mitochondrial OXPHOS, the reversed effects of ursolic acid to doxorubicin resistance were also associated with the regulation of glutamine related amino acid metabolisms [85]. Annonaceous acetogenins (ACGs) are a series of polyketides commonly derived from *Annonaceae* plants with a broad range of biological activities. Cellular metabolic profiling of adriamycin resistant human mammary adenocarcinoma cells revealed that ACGs could enhance the therapeutic effectiveness of adriamycin on mammary adenocarcinoma by influencing multiple metabolic pathways, including glycerophospholipid, proline, arginine, hypotaurine, taurine, glutamate, glutamine, aspartate, and alanine metabolisms [86]. Dietary catechols could overcome the chemoresistance of melanoma A375 cells to cisplatin via inhibiting GST [87]. Oridonin is a tetracyclic diterpenoid component derived from the traditional Chinese herb *Rabdosia labtea*. It was denoted that oridonin could reverse the resistance of pancreatic cancer to gemcitabine through the GST pi-mediated pathway in vitro and in vivo [88]. Capsaicin is the primary constituent of peppers with potential antitumor activity. Garufi et al. showed that capsaicin could increase the sensitivity of chemotherapeutic agents adriamycin and cisplatin through triggering autophagy-mediated mutp53 degradation to provide sufficient amino acid substrates by amino acid recycling [33,89]. Table 2 summarizes the recent studies on combination therapy of chemotherapeutic agents and traditional Chinese medicines to overcome chemoresistance targeting amino acid metabolism.

### 2.4. Nucleotide Metabolism

Compared with normal cells, tumor cells often exhibit abnormal nucleotide metabolism. Nucleotide metabolism is closely associated with tumor initiation and progression, as well as the response to chemotherapy. In terms of purine and pyrimidine syntheses, methotrexate-resistant primary central nervous system lymphoma cell lines exhibited dysregulated expression of thymidylate synthetase, folylpolyglutamate synthetase, and methylenetetrahydrofolate dehydrogenase 1, which further influenced DNA synthesis [90]. Nagel et al. denoted the changes in O^6^-methylguanine DNA methyltransferase contributed to the acquired chemoresistance of temozolomide in glioblastoma multiforme [91]. Phosphoribosyl pyrophosphate synthetase 1 (PRPS1) is a key enzyme to drive the nucleotide biosynthesis pathway. It was reported that PRPS1 was highly and specifically expressed in cisplatin-resistant human breast cancer cell lines, which could be a therapeutic approach to enhance drug responsiveness [92]. Mori et al. suggested that the suppression of thymidine phosphorylase could enhance the drug responsiveness of gastric cancer cells to 5-fluorouracil [93]. Mitochondrial methylenetetrahydrofolate dehydrogenase 2 and its downstream purine synthesis pathway played an important role in the chemoresistance of lung cancer cells to gefitinib [94]. Akhter et al. reported that over-expression of intratumoral thymidylate synthase significantly contributed to the resistance while suppression of dihydropyrimidine dehydrogenase enhanced the sensitivity of 5-FU treatment in oral squamous cell carcinoma [95]. Nicotinamide phosphoribosyltransferase (NAMPT) is an important enzyme for tumor nicotinamide metabolism, which catalyzes the biosynthesis of nicotinamide adenine dinucleotide. A recent study reported that NAMPT could contribute to the resistance of breast cancer to tamoxifen by modulating PKM2 nuclear location [96].

### 2.5. Other Related Metabolisms

**Figure 2:**
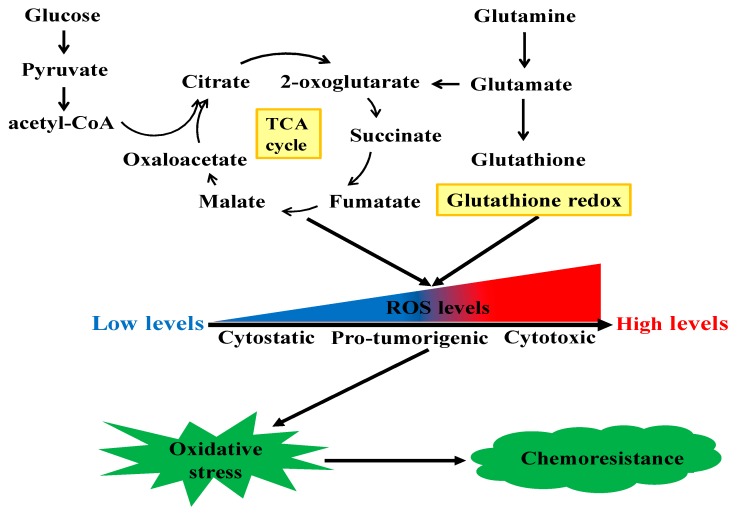
The mechanical illustration of ROS-induced chemoresistance in cancer cells.

In addition to the rewiring of these canonical cell metabolisms associated with chemoresistance, many other metabolic pathways are also reported to be involved in cancer chemotherapy resistance. Besides producing mass-energy for rapid proliferation, chemoresistant cancer cells could also rewire metabolic pathways to maintain their redox homeostasis to confront against oxidative stress. Figure 2 shows the mechanical illustration of reactive oxygen species (ROS)-induced chemoresistance in cancer cells. ROS are a class of highly bioactive molecules to regulate the oxidative state of cells and have dual roles depending on their levels. In normal cells, the levels of ROS are normally low. The enhanced metabolic activity of cancer cells generates relatively high levels of ROS, inducing a pro-tumorigenic state. However, when the levels of ROS increase to the toxicity threshold, a cytotoxic state will occur due to DNA damage. There are various ROS-regulating enzymes to mediate cellular redox homeostasis [97], and dysregulation of these enzymes may alter cellular redox balance and thus result in chemoresistance [98]. For instance, Hosseini et al. reported that the resistance of AML cells to cytarabine was induced by sustaining oxidative stress, which was triggered by high levels of hypochlorous acid catalyzed by the key enzyme myeloperoxidase [99]. Inositol-trisphosphate 3-kinase B (ITPKB) was the critical enzyme which conferred the rapid growth of multiple cisplatin-resistant cancer cells [100]. Mechanistically, inositol 1,3,4,5-tetrakisphosphate produced by ITPKB reduced cisplatin-induced ROS, which facilitated cisplatin-resistant tumor growth. In NSCLC, with short-time exposure of cisplatin, cancer cells developed ROS-mediated metabolism reprogramming via the transcriptional coactivator PGC-1α and thus conferred cisplatin resistance [101]. Heme oxygenase-1 (HO-1) is shown to promote the survival of PDAC cells. The suppression of HO-1 could elevate ROS production and induce apoptosis and thus resensitize PDAC cells to gemcitabine treatment [102]. As an important member of the antioxidant enzyme family to metabolize ROS, glutathione peroxidase (GPX), especially GPX1, is widely expressed in a variety of human cancers. Recently, it was reported that GPX1 could promote the resistance of NSCLC cells to cisplatin through ROS-triggered activation of PI3K/AKT signaling [103]. However, in pancreatic cancer, GPX1 could reverse gemcitabine resistance through the AKT/glycogen synthase kinase 3β (GSK3β)/Snail signaling [104]. Narita et al. found that the inhibition of sestrins 1, a member of highly conserved stress-responsive proteins, could resensitize human maxillary cancer cells to cisplatin treatment via enhancing ROS [105]. Chen et al. showed that estrogen-related receptor alpha, an orphan nuclear receptor, could inhibit the production of ROS and attenuate ROS-induced apoptosis and thus contribute to the methotrexate resistance in osteosarcoma cells [106]. As undifferentiated cancer cells with the properties of self-renewal and differentiation, CSCs contribute to the heterogeneity of tumor tissue and are markedly implicated in the failure of conventional chemotherapies. The epithelial cell adhesion molecule (EpCAM) is a surface marker for CSCs. Yoshida et al. reported that EpCAM was functionally associated with the responses of CSCs to changes in available growth factors in hypo-nutrient conditions [107]. Notably, the redox defense capability of CSCs has been suggested to be responsible for their resistance to conventional anticancer therapies [108]. CD44 is an adhesion molecule of CSCs. CD44 variant stabilizes the light-chain subunit of glutamate-cystine transporter (xCT) and enhances the intracellular glutathione level, which protects cancer cells from redox stress [109]. Interestingly, it was reported that CD44 variant and c-Myc-positive CSCs could attenuate the ROS-induced Wnt signal pathway and induce the therapeutic resistance to the conventionally chemotherapeutic agents [80,110,111].

Traditional Chinese medicine has a long history of treating inflammation-related diseases. Increasing studies revealed that traditional Chinese medicine could regulate cellular ROS levels and thus enhance chemotherapy responsiveness of cancer. For instance, usnea acid is a bioactive secondary metabolite from lichen. Wang et al. revealed that it could reverse the chemoresistance of human chronic myelogenous leukemia to adriamycin via inducing ROS dependent apoptosis [112]. Isofuranodiene is a bioactive sesquiterpene derived from *Alexanders* with a broad spectrum of antitumor properties. A recent study exhibited a synergism between isofuranodiene and temozolomide in glioma cells, and this effect was induced by the ROS-dependent DNA damage [113]. Alpha-hederin is a key saponin separated from *Hedera helix* with various bioactivities. It could enhance the cytotoxic effects of paclitaxel against NSCLC cells via enhancing the intracellular accumulation of ROS [114]. Salvianolic acid B, a water-soluble phenolic compound derived from *Salvia miltiorrhiza*, was revealed to reverse the resistance of human colorectal cancer cells to vincristine via enriching the intracellular ROS amounts [115]. Liquiritigenin, a kind of flavanone commonly derived from *Glycyrrhiza uralensis Fisch*, was demonstrated to exhibit antagonistic effects against gemcitabine-induced capillary leak syndrome in pancreatic adenocarcinoma through suppressing ROS-mediated signaling pathways [116]. Gambogic acid, the prominent active compound obtained from gamboge, could enhance the chemosensitivity of ovarian cancer cells to doxorubicin by inducing ROS-mediated apoptosis [117]. Phenethyl isothiocyanate, derived from common cruciferous vegetables, was reported to induce the apoptotic cell death via ROS dependent pathway in gefitinib-resistant human lung cancer cells [118]. β-elemene, isolated from *Curcuma zedoaria Roscoe*, was shown to enhance the sensitivity of lung cancer cells to cisplatin by increasing intracellular ROS levels and decreasing cytoplasmic glutathione amounts [119]. Isoflavones and biflavonoids from *Ormocarpum kirkii* were reported to induce apoptosis in multiple chemoresistant carcinoma cells through enhanced ROS generation [120]. Phillygenin is a plant-derived tetrahydrofurofuran lignan. He et al. reported that it could inhibit the in vitro and in vivo growth of vindesine-resistant esophageal cancer cells via inducing ROS generation and apoptosis [121]. As a natural product derived from bees, propolis was commonly used for a long time due to its various bioactivities. Herrera et al. reported that Cuban propolis extract and its main ingredient nemorosone could resensitize the chemoresistance of human colon carcinoma cells to doxorubicin. Mechanically, co-treatment induced apoptosis via a pronounced ROS generation [122].

Increasing evidence underscores the essential correlations between gut microbiota and chemotherapy efficacy [123]. Gut microbiota could affect cancer initiation and progression as well as the response to cancer therapy, which is firmly attributable to their intrinsic capacities for drug metabolism and their function on host metabolic homeostasis [124]. Su et al. reported that a polysaccharide derived from the spore of *Ganoderma lucidum* could enhance the sensitivity of breast cancer to paclitaxel by reshaping the gut microbiota and inhibiting tumor metabolism [125]. In detail, the combination treatment of polysaccharide and paclitaxel could restore the gut microbiota dysbiosis triggered by paclitaxel. Notably, the combination treatment enriched the level of *Ruminococcus*, which was markedly negative-correlated with the level of fructose-6-phosphate in the tumor. Table 3 summarizes the recent studies on combination therapy of chemotherapeutic agents and traditional Chinese medicines to overcome chemoresistance targeting other related.

## 3. Perspectives of Metabolic Targeting to Chemoresistance

Based on our systematic review of the underlying metabolic reprogramming mechanisms by which cancer cells develop chemoresistance and traditional Chinese medicines resensitize chemotherapy, the metabolic interference seems to have great potential for cancer therapy. However, despite these attractive observations, many challenges still exist to be fully unraveled. Firstly, metabolic reprogramming during the occurrence of chemoresistance is a complex process, which is co-regulated by multiple factors, such as tumor suppressor genes, oncogenes, and tumor microenvironment [126,127]. Besides, the chemoresistance also exhibits multifactorial and redundant nature. Both the gene and metabolite networks and changes in tumor microenvironment triggering metabolic reprogramming associated with chemoresistance remain to be thoroughly investigated. Secondly, although clinical treatment for cancer therapy has been approved with metabolic interference, its efficacy is still unclear. An inextricable link among cancer epigenetics, cancer immunity, and cancer metabolism has been suggested [12]. Metabolic interference with other modern therapies such as combination of metabolic inhibitors with immune-agonists may have attractive prospects. Thirdly, the majority of currently investigated chemosensitizers for cancer chemotherapy are pure compounds derived from traditional Chinese medicine. Given the “multi-components and multi-targets” manifestations which traditional Chinese medicine always exhibits, investigations of appropriate and relevant formulations from traditional Chinese medicine should also be added. Fourthly, although traditional Chinese medicine has been successfully used as a chemosensitizer, further studies should be added to explore the pharmacokinetic properties both in vitro and in vivo to optimize the best ingredients and dosage for combinatory cancer therapy in the clinic. Finally, the attractive observations of traditional Chinese medicines as chemosensitizers are mostly based on in vitro investigations; more in vivo investigations and further clinical trials will need to be addressed in terms of administration route, poor solubility, and toxicity concerns.

## 4. Conclusions

In this review, we focus on emerging mechanisms underlying rewired metabolic pathways for cancer chemoresistance in terms of glucose and energy, lipid, amino acid, and nucleotide metabolisms, as well as other related metabolisms. In particular, we highlight the potential of traditional Chinese medicine as a chemosensitizer for cancer chemotherapy from the metabolic perspective. The perspectives of metabolic targeting to chemoresistance are also discussed. Once the relationship between metabolic reprogramming and chemotherapy resistance is better understood, we will hopefully expect that the metabolic interference would open up new avenues for overcoming chemotherapy resistance and traditional Chinese medicine as a chemosensitizer would be clinically used for cancer therapy shortly.

## Figures and Tables

**Table 1:** The summary of recent studies on traditional Chinese medicine in combination of chemotherapeutics for overcoming resistance targeting glucose and energy metabolism.

References	Chinese Medicine	Chemotherapy	Cancer	Study	Targeting Metabolic Pathways
25333894	Baicalein	5-uorouracil	Gastric cancer	AGS in vitro	Inhibiting the phosphatase and tensin homolog deleted on chromosome 10 (PTEN)/protein kinase B (Akt)/hypoxia-inducible factor-1alpha (HIF-1α) mediated glycolysis
31211467	Deguelin	Vemurafenib	Melanoma	A2058R and A375R in vitro	Inhibiting oxygen consumption by activated adenosine monophosphate-activated protein kinase (AMPK) signaling
30689992	Trichostatin A	Sunitinib	Renal cell carcinoma	786-O Res in vitro	Triggering intracellular metabolome shifts regarding energy metabolism
30732900	Sodium selenite	Doxorubicin	Bladder cancer	RT-112/D21 in vitro	Altering mitochondrial functions
26987443	Furanodiene	Doxorubicin	Breast cancer	MCF-7/DOX in vitro	Altering mitochondrial functions
30496631	Rhein	Doxorubicin	Liver cancer	SMMC-7721/DOX in vitro	Inhibiting mitochondrial energy metabolism
28785292	Dahuang zhechong pill	Doxorubicin	Liver cancer	SMMC-7721 in vitro	Reducing the adenosine triphosphate (ATP) level by suppressing the key enzymes involved in tricarboxylic acid (TCA) and oxidative phosphorylation (OXPHOS)
29609009	Dahuang zhechong pill	Doxorubicin	Liver cancer	SMMC-7721 in vitro and SMMC-7721 xenografts in mice in vivo	Reducing the ATP level by suppressing the key enzymes involved in TCA and OXPHOS

**Table 2:** The summary of recent studies on traditional Chinese medicine in combination of chemotherapeutics for overcoming resistance targeting amino acid metabolism.

References	Chinese Medicine	Chemotherapy	Cancer	Study	Targeting Metabolic Pathways
30572191	Ursolic acid	Doxorubicin	Breast cancer	MCF-7/ADR in vitro	Regulating the energy metabolism and amino acid metabolism related to glutamine
29750943	Annonaceous acetogenins	Adriamycin	Mammary adenocarcinoma	MCF- 7/Adr cells in vitro	Regulating multiple amino acid metabolism pathways
31339696	Catechols	Cisplatin	Melanoma	A375 cells in vitro	Inhibiting glutathione S-transferase
31410021	Oridonin	Gemcitabine	Pancreatic cancer	PANC-1 and PANC-1/Gem cells in vitro and xenograft tumor model in vivo	Regulating glutathione S-transferase (GST) pi and low-density lipoprotein receptor protein 1 (LRP1)/extracellular signal-regulated kinase (ERK)/c-Jun N-terminal kinase (JNK) signaling
27599722	Capsaicin	Adriamycin and cisplatin	Lung cancer, glioblastoma and breast cancer	H1299, U373 and SKBR3 in vitro	Triggering autophagy mediated mutp53 degradation to provide sufficient amino acid substrates by amino acid recycling

**Table 3:** The summary of recent studies on traditional Chinese medicine in combination of chemotherapeutics for overcoming resistance targeting other related metabolisms.

References	Chinese Medicine	Chemotherapy	Cancer	Study	Targeting Metabolic Pathways
30886864	Usnea acid	Adriamycin	Chronic myelogenous leukemia	K562/ADR Cells in vitro	Inducing reactive oxygen species (ROS) dependent apoptosis
30599912	Isofuranodiene	Temozolomide	Glioma	U87, T98 and U251 in vitro	Causing ROS-dependent DNA damage
30340379	Alpha-hederin	Paclitaxel	Non-small cell lung cancer	NCI-H1299 and NCI-H1650 in vitro	Increasing ROS accumulation
28000873	Salvianolic acid B	Vincristine	Colorectal cancer	HCT-8/VCR cells in vitro	Increasing ROS levels
26028101	Liquiritigenin	Gemcitabine	Pancreatic adenocarcinoma	Panc-1 and HUVECs cells in vitro	Inhibiting ROS-mediated signaling pathways
23436279	Gambogic acid	Doxorubicin	Ovarian cancer	SKOV-3 in vitro	Inducing ROS-mediated apoptosis
29599332	Phenethyl Isothiocyanate	Gefitinib	Lung cancer	NCI-H460 and NCI-H460/G cells in vitro	Inducing apoptotic cell death via ROS dependent pathway
24627125	β-elemene	Cisplatin	Lung cancer	A549/DDP cells in vitro	Increasing intracellular ROS concentration and decreasing cytoplasmic glutathione levels
30836216	Isoflavones and biflavonoids from *Ormocarpum kirkii*	Doxorubicin and geneticin	A panel of 7 carcinomas	Multiple chemoresistant carcinoma cells in vitro	Inducing apoptosis through enhanced ROS generation
30681987	Phillygenin	Vindesine	Esophageal cancer	SH-1-V1 cells in vitro and xenografted mouse model in vivo	Inducing ROS generation and apoptosis
31085307	Cuban propolis and nemorosone	Doxorubicin	Colon carcinoma	LoVo Dox and LoVo in vitro	Inducing apoptosis by a marked ROS production
30619178	Polysaccharide from the spore of *Ganoderma lucidum*	Paclitaxel	Breast cancer	Murine 4T1-breast cancer model in vivo	Suppressing on tumor metabolism with gut microbiota reshaping
